# Nanopore sequencing from formalin-fixed paraffin-embedded specimens for copy-number profiling and methylation-based CNS tumor classification

**DOI:** 10.1007/s00401-024-02731-z

**Published:** 2024-04-20

**Authors:** Ann-Kristin Afflerbach, Anne Albers, Anton Appelt, Leonille Schweizer, Werner Paulus, Michael Bockmayr, Ulrich Schüller, Christian Thomas

**Affiliations:** 1 Department of Pediatric Hematology and Oncology, University Medical Center Hamburg-Eppendorf, Hamburg, Germany; 2 Research Institute Children’s Cancer Center Hamburg, Hamburg, Germany; 3 Institute for Tumor Biology, University Medical Center Hamburg-Eppendorf, Hamburg, Germany; 4 Institute of Neuropathology, University Hospital Münster, Pottkamp 2, 48149 Münster, Germany; 5 Institute of Neurology (Edinger Institute), University Hospital Frankfurt, Goethe University, Frankfurt Am Main, Germany; 6 German Cancer Research Center (DKFZ), German Cancer Consortium (DKTK), Partner Site Frankfurt/Mainz, Heidelberg, Germany; 7 Frankfurt Cancer Institute (FCI), Frankfurt Am Main, Germany; 8 bAIome - Center for Biomedical AI, University Medical Center Hamburg-Eppendorf, Hamburg, Germany; 9 Institute of Neuropathology, University Medical Center Hamburg-Eppendorf, Hamburg, Germany

**Keywords:** CNS tumors, Nanopore sequencing, Formalin-fixed paraffin-embedded

Microarray-based DNA methylation profiling has emerged as a powerful tool for central nervous system (CNS) tumor classification and investigation of diagnostically relevant copy-number alterations such as 1p/19q co-deletion in oligodendroglioma or the +7/−10 signature in glioblastoma [3]. Methylation arrays are well compatible with formalin-fixed and paraffin-embedded (FFPE) derived DNA, but despite these advantages, the most recent release of the EPIC methylation array is still time-consuming and requires processing in batches. Nanopore sequencing has emerged as a rapid and scalable method, enabling direct measurement of methylated cytosines and generation of copy-number profiles, but has been limited to high-quality DNA from native or cryopreserved samples so far [5, 8]. First approaches have been conducted to use nanopore sequencing with FFPE-derived DNA, but they were restricted to specific genes and the detection of point mutations using amplicon sequencing [6, 7]. Here, we demonstrate the possibility of whole genome nanopore sequencing from FFPE-derived DNA for methylation-based classification of CNS tumors and the generation of genome-wide copy-number profiles.

FFPE samples from 40 CNS tumors were retrieved from the archives of the Institutes of Neuropathology Hamburg, Münster, and Frankfurt (all Germany). Methylation array data were available for all cases. After DNA isolation from FFPE material using the Maxwell 16 FFPE Plus LEV DNA Purification Kit or RSC FFPE Plus DNA Kit (Promega), library preparation was performed with the ligation sequencing kit (Oxford Nanopore Technologies, SQK-LSK114) for single samples or the native barcoding kit (SQK-NBD114) for multiple barcoded samples. Libraries were sequenced on MinION (Mk1b/Mk1c), or GridION devices using MinION R10.4.1 flow cells (FLO-MIN114). Libraries from 6 samples were also sequenced on individual Flongle R10.4.1 flow cells (FLO-FLG114). A detailed description of our protocol is available in Supplementary Methods.

**Fig. 1:**
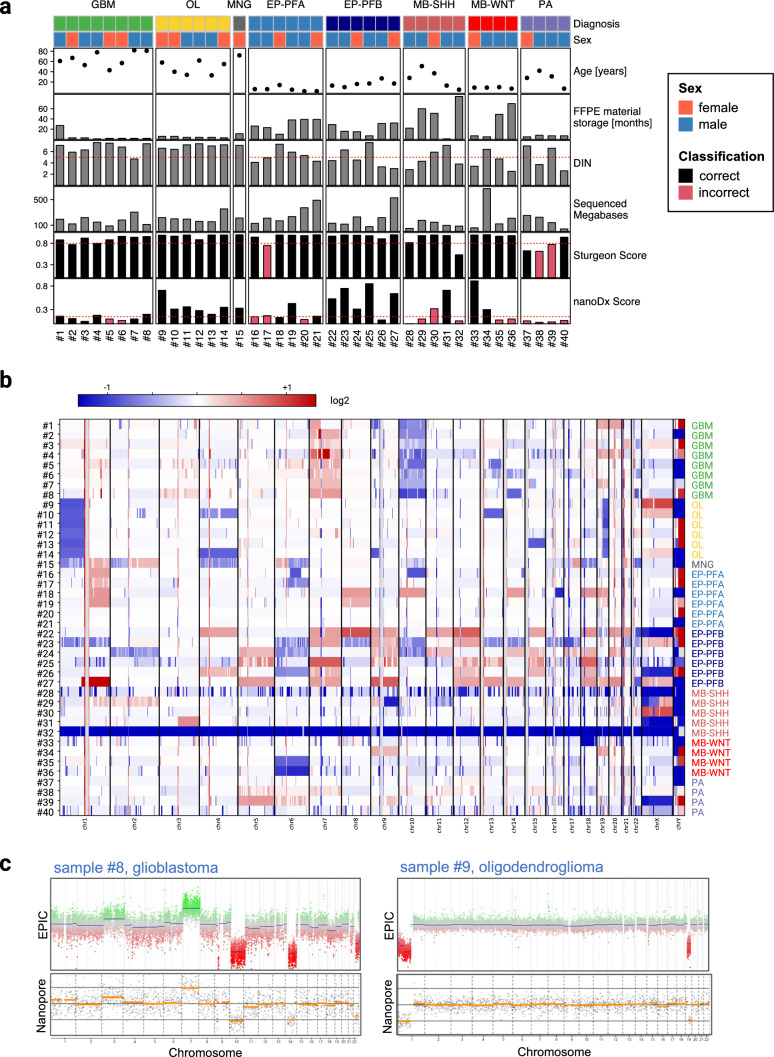
Sample overview and results of FFPE DNA nanopore sequencing analysis. **a** Sample overview of cohort with clinical characteristics as well as analysis results. **b** CNV heatmap of analyzed samples, with losses in blue and gains in red. Samples show expected chromosomal alterations, especially glioblastomas (GBM) with the characteristic +7/−10 signature and co-deletion of Chr 1p and 19q in all oligodendrogliomas (OL). Samples #28 and #32 did not produce enough data for sufficient CNV representation. **c** Comparison of EPIC CNV plots (top) and Nanopore CNV plots (bottom) for samples #8 and #9, showing high concordance between the two methods

Our cohort comprised IDH-wildtype glioblastomas (*n* = 8), oligodendrogliomas (*n* = 6), posterior fossa ependymomas (Group A: *n* = 6, Group B: *n* = 6), medulloblastomas (WNT: *n* = 4, SHH: *n* = 5), pilocytic astrocytomas (*n* = 4), and one meningioma. All samples were analyzed with the Illumina EPIC methylation array as part of routine neuropathology diagnostics (mean calibrated score: 0.97, Supplementary Table 1). The average storage duration of FFPE samples was 19 months (range, 1–84 months). Samples were sequenced at three centers (center A: *n* = 15, center B: *n* = 21, center C: n = 4). On average, sequencing runs produced 205,000 reads (range, 19,700–680,280; Supplementary Table 1) with an average of 201 Mb sequenced per run (range, 42–672 Mb; Fig. 1A, Supplementary Table 1). This resulted in an average human genome coverage of 0.06x. The median fragment length of aligned reads (N50) was 541 bp (range, 279–974 bp) and correlated with DNA integrity values (DIN) as a proxy of DNA quality (*R* = 0.68, *p* < 0.001; Supplementary Fig. 1). Methylation-based analysis of the nanopore sequencing data was performed using the previously published random forest classifier nanoDx [5] and the neuronal network classifier Sturgeon [8]. Whereas the correct methylation class (highest random forest score) using nanoDx was assigned in only 25/40 samples (63%), Sturgeon classified the vast majority of samples correctly (37/40, 93%, Fig. 1A, Supplementary Table 1). Using the recommended thresholds of 0.15 for nanoDx [5] and 0.8 for Sturgeon [8], the classifiers assigned the correct methylation classes in 20 (50%) and 34 (85%) cases, respectively. Of note, all 34 samples with a Sturgeon score ≥ 0.8 were classified correctly. The cohort comprised 16 samples (40%) with poor DNA integrity (DIN values < 5) and significantly longer FFPE storage durations compared to samples with high DNA integrity (28 vs. 5 months, *p* < 0.001, Mann–Whitney U test). Of those, 14/16 (88%) were correctly classified by Sturgeon, suggesting that even low-quality samples are amenable to nanopore-based methylation profiling. Next, we analyzed chromosome-wide copy-number profiles across our samples based on the sequencing data (Fig. 1B). Of note, all IDH-wildtype glioblastomas showed the + 7/−10 signature (sample #1 only 7p gain in accordance with the EPIC CNV profile), and all oligodendrogliomas harbored a co-deletion of Chr 1p and 19q (Fig. 1B, C). Focal copy-number alterations, including high-level amplifications of *EGFR, MDM4, PDGFRA, TERT,* and *CDK6,* as well as homozygous deletions of *CDKN2A/B* were present in 6/8 glioblastomas on EPIC-derived CNV profiling (Supplementary Table 1). However, upon manual inspection of all copy-number profiles derived from nanopore sequencing data, focal alterations were not reliably detectable (data not shown). Next, we sequenced all oligodendrogliomas from our cohort (samples #9—#14) on individual Flongle flow cells with an average of 18 Mb per sample (range, 11—33 Mb, Supplementary Table 2). Of note, 5/6 samples were correctly classified as oligodendrogliomas by Sturgeon and copy-number profiles showed 1p/19q co-deletion in all samples (Supplementary Fig. 2).

Taken together, our study demonstrates the possibility of rapid methylation profiling and copy-number analysis of FFPE specimens using nanopore sequencing. Sturgeon, a neural network-based classifer [8], performed considerably better than the random forest-based classifier nanoDx [5], as observed previously in methylation-based tumor classification [4], whereas the entire cohort was sequenced on MinION flow cells with an average of 201 Mb per sample, even < 10% of the data (average: 18 Mb) produced by low-budget Flongle flow cells (list price: $90) was sufficient for methylation profiling in 5/6 cases and copy-number profiling of all 6 oligodendroglioma samples. However, 6/40 samples (15%) of the entire cohort were below the recommended Sturgeon threshold of 0.8, but longer sequencing durations resulting in a higher CpG coverage could improve methylation-based classification. Current limitations of both nanoDx and Sturgeon include the restriction to methylation classes of the v11b4 training set [2], thus not comprising relevant CNS tumor types such as high-grade astrocytoma with piloid features (HGAP). Methylation profiling employing the Illumina EPIC array necessitates DNA-to-answer turnaround times of 3–4 days, whereas our protocol requires a library preparation time of < 6 h following DNA extraction. Given the possibility of flow cell reuse, adjustable sequencing duration, and barcoding for parallel sequencing of multiple samples (max. five samples in our experiments), nanopore sequencing is highly scalable for neuropathology diagnostic purposes. Moreover, library preparation can be performed with minimal DNA amounts [1]. Due to negligible capital costs for the nanopore sequencing device and minor additional requirements, the workflow is readily applicable to smaller neuropathology labs or lower-infrastructure locations.

### Supplementary Information

Below is the link to the electronic supplementary material.

Supplementary file1 (XLSX 70 kb)

Supplementary file2 (PDF 539 kb)
