# Supplementary material for: Nanopore sequencing from formalin-fixed paraffin-embedded specimens for copy-number profiling and methylation-based CNS tumor classification
Source: Acta Neuropathol. 2024 Apr 20;147(1):74. doi: 10.1007/s00401-024-02731-z (PMC11032293; doi:10.1007/s00401-024-02731-z)
Supplement: Supplementary file 2 — Supplementary file2 (PDF 539 kb) [file 401_2024_2731_MOESM2_ESM.pdf]

Supplementary Methods

Materials: Formalin-fixed paraffin-embedded (FFPE) samples of 40 CNS tumors comprising IDH-wildtype glioblastomas (n=8), oligodendrogliomas (n=6), posterior fossa ependymomas (Group A: n=6, Group B: n=6), medulloblastomas (WNT: n=4, SHH: n=5), pilocytic astrocytomas (n=4), and one meningioma were retrieved from the archives of the Institute of Neuropathology Münster, Hamburg and Frankfurt. The use of biopsy specimens for research upon anonymization was in accordance with local regulations of the University Hospitals Hamburg, Frankfurt and Münster and approved by the Münster ethics committee (2007-420-f-S and 2017-707-f-S).

DNA isolation: Genomic DNA was isolated from FFPE material using the Maxwell 16 FFPE Plus LEV DNA Purification Kit and Maxwell RSC FFPE Plus DNA Kit (Promega). All DNA samples were eluted in 50 µl 1x TE Buffer pH 7.5 (Promega). Genomic DNA was quantified using the QuantiFluor ONE dsDNA System (Promega). DNA integrity (DIN) values and molarity of samples were estimated using the Genomic DNA ScreenTape Assay for the TapeStation (Agilent).

EPIC methylation analysis: DNA isolated from FFPE tissue was subjected to DNA methylation profiling using MethylationEPIC MethylationEPIC v1 or v2 BeadChip array using materials and protocols provided by the manufacturer (Illumina Inc., San Diego, CA).

Library preparation and Nanopore sequencing: Single library preparation was performed using the Ligation Sequencing Kit SQK-LSK114 (Oxford Nanopore Technologies) with 155-260 fmol genomic DNA. Multiplex library preparation for samples #4 - #8 was performed using the Native Barcoding Kit SQK-NBD114.24 with up to five genomic DNA samples and 400 ng input for each sample. Quantification steps throughout the Nanopore protocols were performed using the gDNA ScreenTape Assay for the TapeStation (Agilent) and the recommended Qubit (Qubit 3.0, Invitrogen) quantification steps. Libraries were equilibrated to 26 – 61 fmol and sequenced on R10.4.1 flow cells using either MinION Mk1B, Mk1C or GridION devices (Oxford Nanopore Technologies). Libraries from samples #9 - #14 were additionally sequenced on individual Flongle R10.4.1 flow cells (FLO-FLG114).

Data analysis: Data was demultiplexed with MinKNOW when needed. Basecalling for methylation analysis was performed with Guppy 4.4.2 for nanoDx [3] analysis. For Sturgeon [6] analysis, the high accuracy model (hac@v4.3.0) of Dorado 0.4.3 was used. The nanoDx random forest classifier was employed as described previously [1]. Prior to Sturgeon classification, the

basecalled data was aligned to the T2T reference genome (CHM13v2) within Dorado. Modkit 0.2.2 was then used to adjust 5hmC modification calls to 5mC calls. CNV analysis based on the sequence data was carried out as described before with window sizes set to 500,000 bp [1]. The CNV heatmap was calculated with a window size of 500 kbp and visualized using CNVkit [5]. The mean read length was calculated using NanoPlot [2]. For further visualization of CNV plots, ACE [4] was used with a 1000kbp window.

References

1. Afflerbach A-K, Rohrandt C, Brändl B, Sönksen M, Hench J, Frank S, Börnigen D, Alawi M, Mynarek M, Winkler B, Ricklefs F, Synowitz M, Dührsen L, Rutkowski S, Wefers AK, Müller F-J, Schoof M, Schüller U (2024) Classification of Brain Tumors by Nanopore Sequencing of Cell-Free DNA from Cerebrospinal Fluid. Clin Chem 70:250–260

2. De Coster W, Rademakers R (2023) NanoPack2: population-scale evaluation of long-read sequencing data. Bioinformatics 39. doi: 10.1093/bioinformatics/btad311

3. Kuschel LP, Hench J, Frank S, Hench IB, Girard E, Blanluet M, Masliah-Planchon J, Misch M, Onken J, Czabanka M, Yuan D, Lukassen S, Karau P, Ishaque N, Hain EG, Heppner F, Idbaih A, Behr N, Harms C, Capper D, Euskirchen P (2023) Robust methylation-based classification of brain tumours using nanopore sequencing. Neuropathol Appl Neurobiol 49:e12856

4. Poell JB, Mendeville M, Sie D, Brink A, Brakenhoff RH, Ylstra B (2019) ACE: absolute copy number estimation from low-coverage whole-genome sequencing data. Bioinformatics 35:2847–2849

5. Talevich E, Shain AH, Botton T, Bastian BC (2016) CNVkit: Genome-Wide Copy Number Detection and Visualization from Targeted DNA Sequencing. PLoS Comput Biol 12:e1004873

6. Vermeulen C, Pagès-Gallego M, Kester L, Kranendonk MEG, Wesseling P, Verburg N, de Witt Hamer P, Kooi EJ, Dankmeijer L, van der Lugt J, van Baarsen K, Hoving EW, Tops BBJ, de Ridder J (2023) Ultra-fast deep-learned CNS tumour classification during surgery. Nature 622:842–849

Supplementary Figures

Supplementary Figure 1

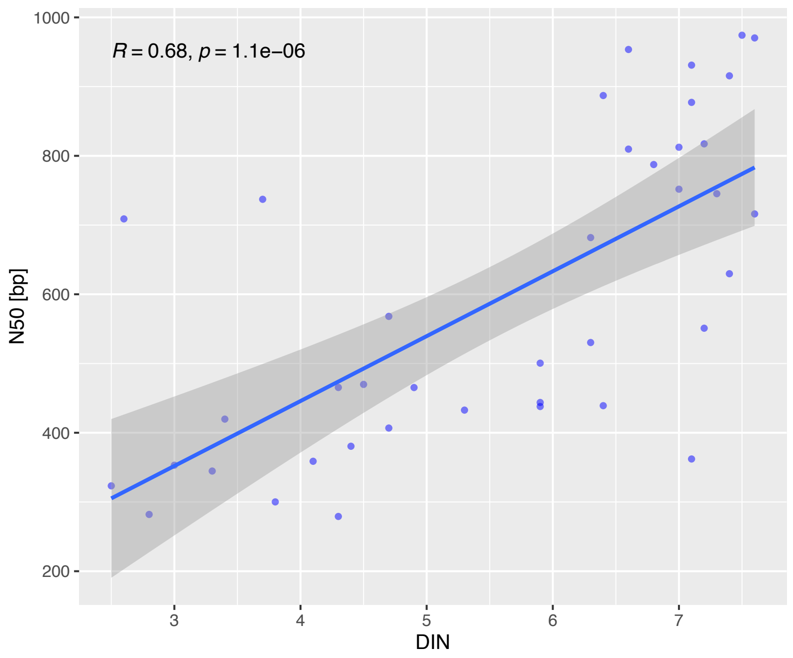

Supplementary Figure 1: Correlation of DIN values with N50. DIN values of samples show high correlation with median sequencing length (R=0.68, p<0.001).

Supplementary Figure 2

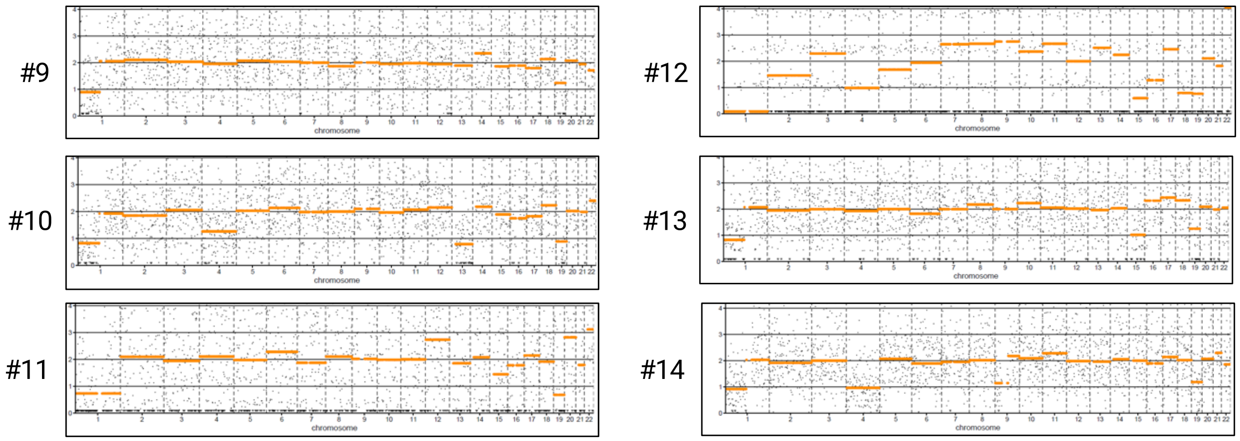

Supplementary Figure 2: CNV profiles of oligodendrogliomas sequenced on Flongle flow cells. Samples #9 - #14 were each sequenced on individual Flongle flow cells. Genome-wide chromosomal copy-number profiles show reliable co-deletion of Chr 1p and 19q in all samples, although #12 (the sample with the lowest sequencing amount of 10.9 Mb) was considerably more noisy compared to all other samples.
